# I Publish in I Edit? - Do Editorial Board Members of Urologic Journals Preferentially Publish Their Own Scientific Work?

**DOI:** 10.1371/journal.pone.0083709

**Published:** 2013-12-27

**Authors:** Jens Mani, Jasmina Makarević, Eva Juengel, Hanns Ackermann, Karen Nelson, Georg Bartsch, Axel Haferkamp, Roman A. Blaheta

**Affiliations:** 1 Department of Urology, Johann Wolfgang Goethe-University, Frankfurt am Main, Germany; 2 Institute of Biostatistics, Johann Wolfgang Goethe-University, Frankfurt am Main, Germany; 3 Department of Vascular and Endovascular Surgery, Johann Wolfgang Goethe-University, Frankfurt am Main, Germany; Humboldt-Universität zu Berlin, Germany

## Abstract

Scientists who are members of an editorial board have been accused of preferentially publishing their scientific work in the journal where they serve as editor. Reputation and academic standing do depend on an uninterrupted flow of published scientific work and the question does arise as to whether publication mainly occurs in the self-edited journal. This investigation was designed to determine whether editorial board members of five urological journals were more likely to publish their research reports in their own rather than in other journals. A retrospective analysis was conducted for all original reports published from 2001–2010 by 65 editorial board members nominated to the boards of five impact leading urologic journals in 2006. Publications before editorial board membership, 2001–2005, and publications within the period of time as an editorial board member, 2006–2010, were identified. The impact factors of the journals were also recorded over the time period 2001–2010 to see whether a change in impact factor correlated with publication locality. In the five journals as a whole, scientific work was not preferentially published in the journal in which the scientists served as editor. However, significant heterogeneity among the journals was evident. One journal showed a significant increase in the amount of published papers in the ‘own’ journal after assumption of editorship, three journals showed no change and one journal showed a highly significant decrease in publishing in the ‘own’ journal after assumption of editorship.

## Introduction

Academic publishing occurs in a situation where intellectual, financial, and occasional political interest may enter into the publishing process [1]. Hearsay intimates that scientists who are members of an editorial board preferentially publish their scientific work in the journal where they serve as editor. Although editorial board members, as academically active clinicians and researchers, are allowed to publish in their ‘own’ journal, a “type of camaraderie” [2] has been proposed to exist which may facilitate the review process.

National and international reputation and academic standing all depend on an uninterrupted flow of published scientific work. Co-authorship, while fellow staff members climb the medical career ladder, is likewise important since clinical and scientific influence and an extended network stem from fellow associates being promoted. Finally, medical research financing is often determined by the sum of publications, particularly in high impact journals. With this in mind, preferential publishment of scientific work in the journal where the scientist serves as editor might open the way to scientific and private misconduct with considerable effects on the scientific community as a whole. A former editor has postulated that publication policy is biased [3], while journal editors maintain that fair standards apply to their journals’ peer review processes. Notwithstanding, “publication bias” is a broadly perceived preconception.

Unfortunately, most journals do not have a written policy, readily available to their readers and authors, regarding manuscript submission by editorial board members [2]. Analysis of journal transparency has revealed that the majority of journals are not explicit enough in their “instructions for authors” [4] and the lack of transparency may promote accusations of ‘insider’ favoritism [2], [5]. Haivas et al. have noted that “although many journals now publish authors’ financial conflicts of interest, and reviewers are asked to declare if they have a conflict of interest with regard to individual manuscripts, little is known about editors’ conflicts of interest and the mechanisms to manage them” [6].

Most editors do not release information regarding the evaluation of manuscripts submitted by their own editorial board members [7]. It is, therefore, not surprising that reports dealing with self-publication practices of journal editors are sparse. Indeed, it is unclear, whether editorial board members tend to change their publication behaviour before and after acquiring journal editorship. To shed more light on this, the present investigation was designed to explore whether editorial board members of selected urologic journals were more likely to publish their research reports in their own journal rather than in other journals.

## Methods

### Analysis Strategy

A retrospective analysis was conducted for all original reports published from 2001–2010 by 65 editorial board members nominated to the boards of five leading (according to impact factor) urologic journals in 2006. Journal search was based on the subject category “Urology and Nephrology” in the ISI Web of Knowledge Journal Citation Reports. Those journals were selected which cover a broad field of urology, publishing original clinical, original experimental, review and commentarial articles. Journals exclusively focusing on a specific urologic field were not included. In 2006, the editorial boards were changed for all five journals, allowing an analysis of publishing policy before and after the change. The journals were European Urology, Urology, World Journal of Urology, The Journal of Urology and British Journal of Urology International (BJU). Reviews and editorial comments were neglected. All journals affirm a strict ethical code pertaining to the integrity of the scientific reports submitted. Publications were searched by author name. To exclude false attribution due to homonyms, author names and affiliations were cross-checked in each publication and compared to the editorial board members list.

Publications before editorial board membership (2001–2005, “pre-editorial period”) and publications within the period of time as an editorial board member (2006–2010) were identified using the Thomson Reuters (ISI) Web of Knowledge and the PubMed database supplied by the U.S. National Library of Medicine - National Institutes of Health. The following was determined:

1. Publications in the edited (‘own’) journal.
2. Publications as first or last author in the edited (‘own’) journal.
3. Publications in other urology/nephrology journals with defined impact factor.
4. Publications in all other medical journals.

The impact factors of the journals were also recorded over the time period 2001–2010 to see whether a change in impact factor correlated with publication policy.

### Statistics

Since the number of published articles almost doubled in the editorial period compared to the pre-editorial period relative percent was used to compare where publishing occurred before and during editorship. Box- and whisker plots showing smallest and largest observation, median, 25th and 75th percentile values were prepared. Odds ratios were calculated with Wilcoxon matched pairs test. Pearson correlation coefficients with p values <0.05 were considered significantly different.

A meta-analysis was performed to obtain a pooled estimate of publication effect (PE), to which the impact factor belongs. Calculating the publication effect was done to find out whether there is an association between publishing in ‘own’ journal and impact factor. The association between journal-specific publications and impact factor was calculated as a weighted average of journal-specific estimates (Hodges-Lehmann) and the delta of impact factor (Δ 2010-2001). The Hodges-Lehmann estimate of the effect size and Tukey 95% confidence intervals (CI) for the effect size of the publication in the “own” journal compared to all publications were calculated using the Wilcoxon matched pairs test. The five journals were analyzed jointly using a random-effects model, taking heterogeneity among studies into account in addition to within-study variance. The percentage variability of the pooled PE attributable to heterogeneity between studies was quantified using the I2 statistic.

Statistical work was conducted using the BiAS for windows statistical software program (Version 9.12) and The R Project for Statistical Computing (Version 2.13.1).

## Results

Sixty-five contributors to five leading urologic journals (World Journal of Urology n = 11, Urology n = 3, BJU n = 17, Journal of Urology n = 14, European Urology n = 20), who joined editorial boards in 2006 were identified.

A total of 4645 articles were published by the 65 authors over the time period from 2001 to 2010. 1800 articles were published in the time period 2001 to 2005, before the authors assumed editorship, and 2845 in the time period from 2006–2010, when the authors had assumed editorship.

**Figure 1:**
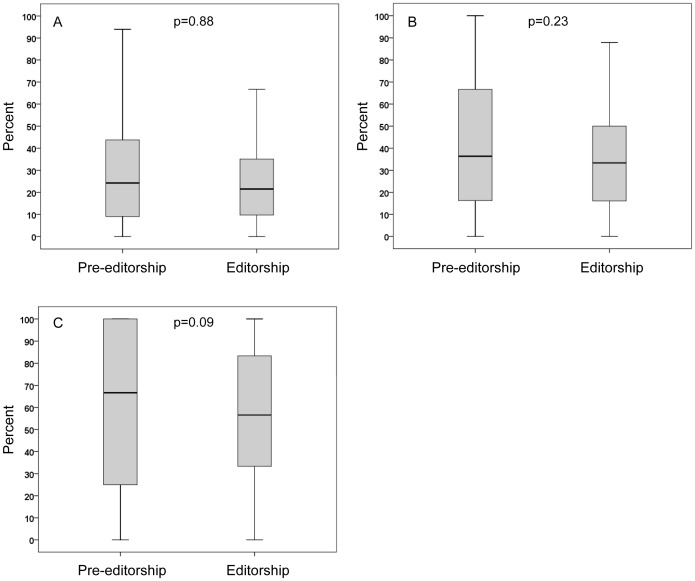
Evaluation of publishing behavior. 1A: Articles in 5 leading urologic journals from 65 authors before (pre-editorship) and later during editorship as a percentage of all articles published by these authors. 1B: Articles in 5 leading urologic journals from 65 authors before (pre-editorship) and later during editorship as a percentage of all articles published in urologic/nephrologic journals by these authors. 1C: Percent of articles in 5 leading urologic journals from 65 authors with first or last co-authorship before (pre-editorship) and later during editorship.

Percent of publications in the ‘own’ journal, compared to all publications, was not significantly different (p = 0.88) during pre-editorship and during editorship (Figure 1A). Likewise, the percent of publications in the ‘own’ journal, compared to publications in all urologic/nephrologic journals was not significantly different (p = 0.23) during pre-editorship and during editorship (Figure 1B). Figure 1C shows that the percent of first or last authorships during pre-editorship and during editorship were not significantly different in the ‘own’ journal (p = 0.09).

**Figure 2:**
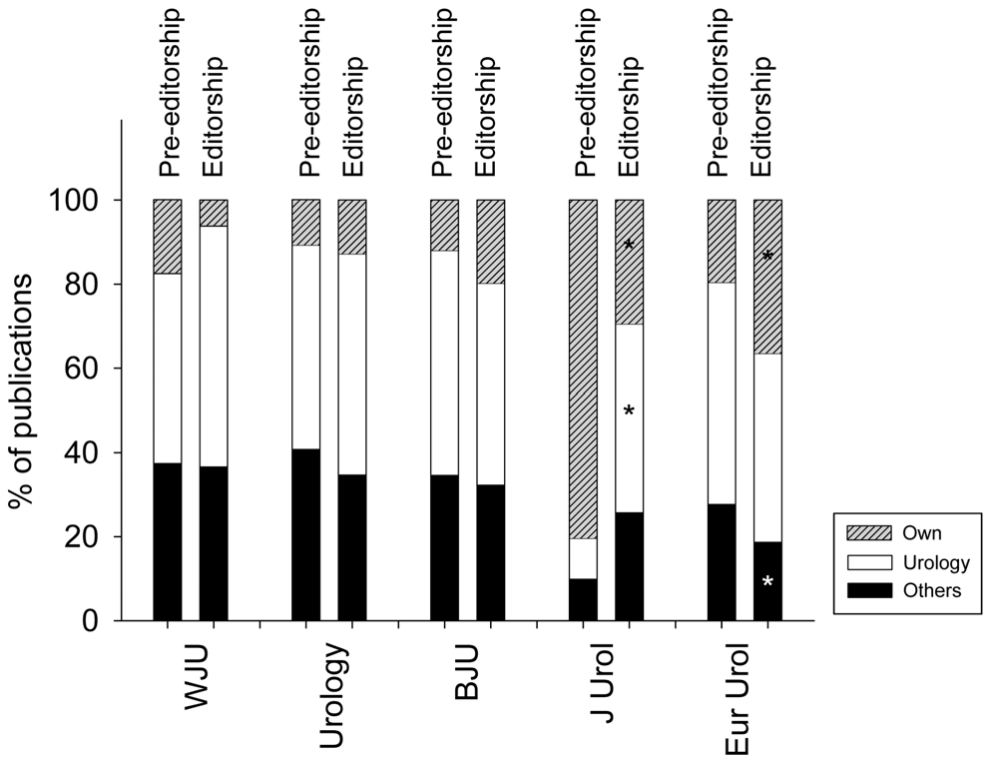
Distribution of published articles from 65 authors before (pre-editorship) and during editorship of 5 selected urologic journals. *indicates significant change compared to pre-editorship. Own = journal pre-editorship and later editorship. Urology = urologic/nephrologic journals. Others = all journals excluding ‘own’ journal and urologic/nephrologic journals.

The relative percent distribution of research articles from 65 authors in each of five urological journalsis demonstrated in Fig. 2, comparing % of published articles pre-editorship with published articles during editorship. There is no significant change in the publication patterns of the editorial board members of World Journal of Urology, Urology and BJU pre-editorially and during editorship. In Journal of Urology there was a significant decrease (p<0.004) from 80 to 30% of articles published in that journal (compared to total amount published) when comparing the time period before editorship with editorship. A significant increase (p<0.001) from 10 (pre-editorship) to 45% (editorship) was observed in published articles in urologic/nephrologic journals other than the self-edited Journal of Urology. In European Urology there was a significant increase (p<0.005) from 20 (pre-editorship) to 36% (editorship) of articles published in that journal (compared to total amount published). A significant decrease (p<0.01) from 28 (pre-editorship) to 19% (editorship) was observed in published articles in journals other than urologic/nephrologic journals.

**Figure 3:**
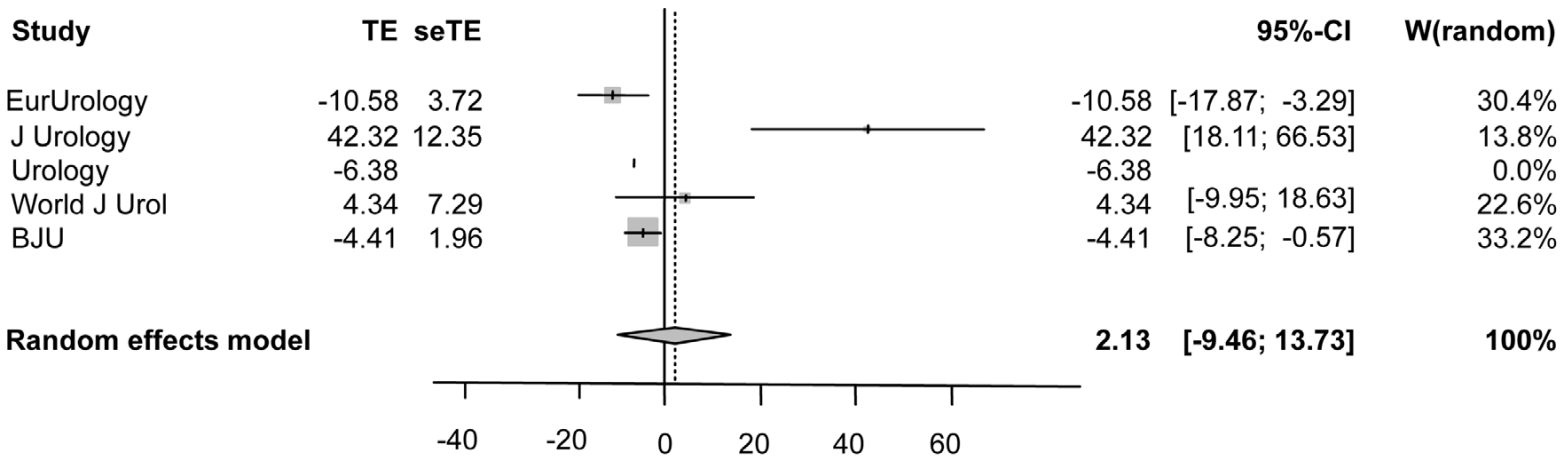
Meta-analysis (forest plot) of publication effects considering change in impact factor from 2001–2010 and the 95% confidence interval of the ratio of editorship/all publications. TE = treatment effect, i.e. publication effect. seTe = standard error of treatment effect, i.e. standard error of publication effect. ◊ = overall effect.

The intra-journal change in publication habits pre-editorship to editorship is also shown in Fig. 3 (which incorporates impact factor) with a significant decrease in publication in the ‘own’ journal after assumption of editorship with a PE of 42.32 (95% CI: 18.11–66.53) for one journal. In another journal a significantly increased PE of 10.58 (95% CI: −17.87–3.29) was apparent after assumption of editorship. The random effects model of the five journals showed an overall publication effect of 2.13 (95% CI: −9.46–13.73, p = 0.7181), with evidence for significant heterogeneity between journals (I2 Statistic = 83.3%, p = 0.0003).

## Discussion

Allegations of unfair advantage concerning publication in the same journal to which scientists serve on editorial boards abound. Journals, in turn, deny favoritism. Reports with different approaches and statistical methods have been published concerning these allegations. The approach used by Luty et al. to decide whether preferential publication of editorial board members exists was to compare the proportion of original research reports in the ‘own’ journal (7.7%) with the proportion in that journal authored by a member of another journal’s editorial board (2.8%) in the same subspecialty [7]. Luty et al. conclude that, in the year 2006, 14 of the 20 journals surveyed showed a statistically significant excess of publications from the journal’s own editorial board [7]. Bošnjak et al. investigated the publishing goals of 256 Croatian editors of 180 Croatian journals over a period of 4 years and found that only 18 of those editors published 5 or more articles in their own journals [8]. They concluded that the majority of editors did not misuse their own journal for scientific publication [8].

The present approach was designed not only for an overall evaluation of editors’ publishing goals in five urological journals, but also to evaluate the publishing practices of editorial board members belonging to each individual journal. To do so, the amount of published articles pre-editorship over a period of five years was compared with the amount of articles post-editorship over a period of five years in the edited journal. The amount was expressed in percent of all published articles in all urologic or nephrologic journals to relativize the expected increase in publishing that occurs as a scientist’s carrier advances.

In the present statistical analysis, referring to the degree to which editorial board members preferentially published their own work in the five investigated journals, an increase in self publishing was not found. However, considerable heterogeneity in the publishing policy of the individual journals was apparent. In one journal there was a significant increase in editorial board members preferentially publishing their own work. In another journal there was a significant decrease, indicating that the editorial members of this journal preferentially published their work in other journals after assuming editorship. In the other three journals assumption of editorship did not influence the percentage of articles published in the ‘own’ journal, compared to the articles published in urologic or nephrologic journals, where editors of urologic journals would logically publish.

To investigate whether there might be an association between journal-specific publications and impact factor, an estimate of publication effect was carried out in the present investigation. Indeed, in the only journal showing a significant increase in editorial board members preferentially publishing their own work, an increase in the impact factor from 2.3 in 2001 to 8.8 in 2010 was noted. The bulk of the increase was noted during the period 2005–2010, corresponding to the period of assumed editorship. Nevertheless, illegitimate impact factor boosting cannot be inferred from the data presented here since an analysis of citation and self-citation was not carried out.

The other investigated journals showed much smaller growths in their impact factors (average: 2.1±1.0 in 2001 to 3.0±0.8 in 2010). Results from a study by Pagel and Hudetz, who investigated publishing goals of editorial board members from 10 anesthesia journals indicate that editorial board members of those journals with higher impact factors have higher h-indexes [9]. The h-index measures the relative quality of an investigator’s collective body of work [10]. However, this quality is based on the number of citations in peer–reviewed articles, which can be influenced by self-publishing and self-citation. Direct influence has been described by Schiermeier [11] whereby a relatively high impact factor corresponded to a high rate of self-citation and self-publication of a particular editor, upon whose retirement the journal’s impact factor was halved.

Although the present investigation incorporated only 5 journals, it concords with the findings described by Luty et al. and Bošnjak et al. [7], [8]. Both Luty et al. and Bošnjak et al. report favoritism towards publication in the ‘own’ journal, Luty et al. in the majority of investigated journals and Bošnjak et al. in a minority. However, both studies found journals where favoritism did not occur. The present study fits this heterogeneous pattern and additionally shows an association between favoritism and impact factor.

Editorial board members are academically active and are, therefore, committed to publishing. Occupying a position on an editorial board reflects high standing within one's specialty and it is plausible that an editorial board member might prefer to publish in the ‘own’ journal, which optimally reflects the field of expertise. An author-editor might also consider publishing in the ‘own’ journal as a sign of loyalty to the journal. ‘Self-publishing’ in a high-ranking journal might be particularly tempting, since the impact factor plays such an important role in the scientific community [12]. Still, where an outstandingly high impact factor corresponds to editors preferentially publishing in their ‘own’ journal, suspicion will inevitably arise that self-publication may be linked to the impact factor.

There is no doubt that editorial integrity is the sine qua non for any academic journal, and editors undertake much effort to avoid corruption [2]. However, there is no distinct line between ethical and unethical behaviour [13]. Developing ethical guidelines for publishing in scientific journals might offer a framework to handle manuscripts from editorial board members. Indeed, the associations ICMJE (International Committee of Medical Journal Editors), COPE (Committee on Publication Ethics) and WAME (World Association of Medical Editors) have already formulated a protocol regarding authors’ conflict of interest, sponsoring, authorship, peer review, plagiarism, advertising and other potential ethical conflicts [5]. Although this protocol provides an excellent platform for the journals to establish their own standards [14], only few journals offer transparency concerning editors’ conflict of interest [6]. An analysis of the top ten peer-reviewed medical journals showed that only four of them had accessible conflict of interest policy directives including editors [5]. In another survey of 37 journals, only 19 of 30 responders considered it important to declare editors’ conflict of interest. Half of these had a policy to deal with the issue, which was “internal” and “often vague” [6], [15].

In order to set better standards, Graf and coworkers have recently developed a “best practice” guideline for editors and editorial board members [1]. Their suggestion is based on the argument that editorial board members, independently of whether they act as authors or reviewers, are often uncertain what to declare as conflict of interest or fail to recognize that they have competing interests [15]. Particularly, personal conflicts, which may be as far reaching as financial conflicts, are difficult to define [16]. In fact, some conflicts of interest are unavoidable, may not be unethical and may not involve wrongdoing [2].

The results presented here demonstrate a swing from significant increase over no change to a highly significant decrease in the amount of published papers in the ‘own’ journal before and after assumption of editorship. Submitting a manuscript to the ‘own’ journal is not unethical per se as long as the evaluation process for editors undergoes the same restrictive procedure as for non-editors. Journals could reevaluate and improve their review standards to disclose interests “that might appear to affect their ability to present or review data objectively” [15]. Both authors and reviewers may need to declare all personal or cooperative relationships. Graf et al. have recommended excluding editorial board members from publication decisions where an editor or board member is an author [1]. Blinded reviewing might also facilitate optimal evaluation. All these measures cannot absolutely prevent all editorial favoritism. However, delivering a readily available journal concept of editorial conflict of interest to the scientific community might help to maintain and improve journal reputation.
